# Progesterone receptor variants and hormone therapy use in postmenopausal endometrial polyps: a case-control study

**DOI:** 10.1016/j.clinsp.2026.101216

**Published:** 2026-10-15

**Authors:** Milton Wajman, Marcos Tcherniakovsky, Marina Martinelli Sonnenfeld, Marta Maria Arcoverde Cavalcanti Meniconi, Marília Franco de Oliveira, Thomas Moscovitz, Rogers Camargo Mariano da Silva, César Eduardo Fernandes, Emerson de Oliveira

**Affiliations:** a Video Endoscopy and Endometriosis Section of The Gynecology Department, Centro Universitário Faculdade de Medicina do ABC, Santo André, SP, Brazil; b Department of Obstetrics and Gynecology, Centro Universitário Faculdade de Medicina do ABC, Santo André, SP, Brazil; c Gynecology Department, Centro Universitário Faculdade de Medicina do ABC, Santo André, SP, Brazil; d Urogynecology Section of The Gynecology Department, Centro Universitário Faculdade de Medicina do ABC, Santo André, SP, Brazil

**Keywords:** Endometrial polyps, Gynecology, Hormone therapy, Polymorphism, Risk factor

## Abstract

**Objective:**

To investigate whether Progesterone Receptor (PGR) variants +331G/A (rs10895068) and V660L (rs1042838, a PROGINS proxy) are associated with Endometrial Polyps (EP) in postmenopausal women undergoing hysteroscopy.

**Methods:**

This exploratory case-control study included 236 histopathologically confirmed EP cases and 73 hysteroscopy-negative controls from the same institution and period, unmatched. All eligible hysteroscopy-negative women during the study period were included, with no declinations. Genotyping of +331G/A and V660L was performed by PCR-HRM. Genetic comparisons used two-sided tests and multivariable logistic regression, with minimum detectable odds ratios estimated for 80% power at α = 0.05. Hormone Therapy (HT) and tamoxifen use were recorded as binary variables reflecting current use at hysteroscopy.

**Results:**

Genotype distributions did not differ between groups for +331G/A (*p* = 0.245) or V660L (*p* = 0.435); call rates were 100% for both variants. Neither +331G/A nor V660L carrier status was significantly associated with EP after adjustment (aOR = 0.43, 95% CI 0.17–1.09, *p* = 0.074; and aOR = 0.59, 95% CI 0.31–1.11, *p* = 0.100, respectively). Current HT use was inversely associated with EP (aOR = 0.15, 95% CI 0.06–0.40; *p* < 0.001), though regimen data were unavailable. Genetic associations remained non-significant in the model excluding HT.

**Conclusion:**

Neither PGR variant was significantly associated with EP. The sample could detect only relatively large effects, so smaller genetic associations cannot be excluded. The inverse association with binary HT exposure is hypothesis-generating rather than causal, given incomplete exposure characterization and the possibility of selection or residual confounding.

## Highlights

• PGR +331G/A and V660L were not statistically associated with endometrial polyps.
• The sample could detect only relatively large genetic associations.
• Current hormone therapy use was inversely associated with polyp status.
• HT formulation, dose, route, duration, schedule, and indication were not collected.
• The HT association is hypothesis-generating and does not support causal inference.

## Introduction

Endometrial Polyps (EP) are common benign focal overgrowths of the endometrium and are frequently identified in women undergoing uterine cavity evaluation, including postmenopausal patients assessed for bleeding or endometrial thickening.^1^, ^2^, ^3^ EP may be symptomatic or incidental, and their clinical relevance in postmenopausal women relates to bleeding, recurrence, and the need to exclude concomitant endometrial pathology.^1^^,^^4^^,^^5^

The pathogenesis of EP is multifactorial and remains incompletely understood. Current evidence supports a model of dysregulated endometrial proliferation influenced by hormonal signaling, particularly an imbalance between estrogenic stimulation and progesterone-mediated counter-regulation, together with local endometrial factors.^1^^,^^6^, ^7^, ^8^ At the tissue level, altered Estrogen Receptor (ER) and Progesterone Receptor (PR) expression has been described in EP, reinforcing the plausibility of hormone receptor biology in polyp development.^7^ Clinical correlates reported across studies include age and metabolic comorbidities such as obesity, diabetes mellitus, and hypertension, although findings are heterogeneous across cohorts.^9^, ^10^, ^11^

Exogenous hormone exposure has also been implicated in endometrial proliferative changes. Hormone Therapy (HT) regimens may differentially influence endometrial growth depending on the balance between estrogen and progestogen components and their mode of administration.^1^^,^^12^ However, the relationship between HT use and EP occurrence in postmenopausal women remains incompletely defined, with heterogeneous findings across clinical settings.

Given the central role of progesterone signaling in endometrial differentiation and growth restraint, genetic variation in the Progesterone Receptor Gene (PGR) has been proposed as a plausible susceptibility factor for endometrial proliferative conditions. The PROGINS haplotype, commonly proxied by the exon 4 variant V660L (rs1042838), has been associated with altered progesterone responsiveness in experimental models and has been investigated in gynecologic phenotypes with inconsistent results across populations.^13^, ^14^, ^15^ In addition, the functional PGR promoter variant +331G/A (rs10895068) has been linked to altered receptor expression and has been studied in hormone-related endometrial outcomes.^16^^,^^17^ However, evidence specifically linking these PGR variants to EP in postmenopausal women remains limited, and available studies are few and often underpowered. Hereafter, the assayed exon 4 variant is referred to as V660L, not as the complete PROGINS haplotype.

Therefore, the primary objective of this study was to investigate whether the progesterone receptor variants +331G/A and V660L are associated with the presence of EP in postmenopausal women undergoing hysteroscopy. As a secondary objective, we compared available clinical characteristics between women with and without EP.

Given the clinical relevance of exogenous hormone exposure in EP pathogenesis, we also explored the association between HT use and EP status.

## Material and methods

### Study design

This was an exploratory observational case-control study based on data collected at Hospital Estadual Mário Covas, Santo André, São Paulo, Brazil. The study included 309 female patients who underwent diagnostic hysteroscopy between March 2018 and January 2021.

The EP+ group comprised postmenopausal women with histopathologically confirmed EP after hysteroscopic polypectomy (n = 236), whereas the EP- group comprised postmenopausal women with a normal uterine cavity at diagnostic hysteroscopy (n = 73). Controls were not individually matched to cases. All eligible women with a normal hysteroscopy during the study period were included, and no invited participant declined. Both groups were evaluated at the same institution during the same period. Hysteroscopy was performed only for clinical indications, principally postmenopausal bleeding or endometrial thickening/suspected intracavitary abnormality on ultrasonography. The distribution of individual referral indications was not recorded for the study, and a numerical screening flow diagram could not be reconstructed. The EP- group therefore represents clinically evaluated hysteroscopy-negative controls rather than population-based controls.

Data were extracted from medical records, including age, systolic and diastolic blood pressure, BMI, diabetes mellitus, smoking status, tamoxifen use, HT use, serum E1 and E2 measurements, and genotypes for +331G/A and V660L. HT and tamoxifen use referred to current use at the time of hysteroscopy and were recorded as binary variables. HT was not suspended for the procedure. Information on HT formulation, dose, route, duration, schedule, and indication was not collected. Current tamoxifen use was for breast cancer; dose, duration, cumulative dose, and treatment timing were not collected.

All selected patients were informed about the study and signed an informed consent form. The study protocol was previously submitted and approved by the Research Ethics Committee of the Faculdade de Medicina do ABC (CAAE 56992016.8.0000.0082).

### Inclusion and exclusion criteria for research participants

The inclusion criteria for the group of patients with EPs were postmenopausal patients, with an indication for polypectomy, who underwent polypectomy by video hysteroscopy between March 2018 and January 2021, with diagnostic confirmation of an EP in the anatomopathological analysis. The exclusion criteria were patients who were in the menacme period, and patients who did not have the clinical conditions to undergo the surgical procedure within the specified period.

In the EP- group the inclusion criteria were postmenopausal patients presenting with normal uterine cavity confirmed by diagnostic video hysteroscopy examination carried out between March 2018 and January 2021. The exclusion criteria were: menacme patients; patients who had a previous history of polypectomy via video hysteroscopy and patients who had no indication for a diagnostic hysteroscopy exam.

### Peripheral venous blood collection

Venous blood samples were collected in both groups via peripheral access in two tubes of 5 mL each, this being the only non-routine intervention for the patients included in the study. Blood samples were collected using a vacutainer containing EDTA in both groups and sent to an external laboratory for DNA extraction and subsequent analysis of polymorphic variants +331 and V660L.

### Molecular analysis

We evaluated the PGR promoter variant +331G/A (rs10895068) and the exon 4 variant V660L (rs1042838), which is commonly used as a marker that proxies the PROGINS haplotype.

The PCR High Resolution Melting (PCR-HRM) technique was used to detect gene sequences based on the temperature difference necessary for the dissociation of the double strand of DNA into a single strand, using saturating fluorescent dyes (EvaGreen), which intercalates with double-stranded DNA. After PCR, the DNA is denatured, the dyes released, and the fluorescence is captured, generating a dissociation curve. Data analysis and interpretation are performed by the equipment software. The HRM protocol was performed according to the manufacturer's instructions. Briefly, final volumes of 25 µL, Type-it HRM PCR (QIAGEN, Brazil), each pair of primers (5 μM) (Table 1),^18^^,^^19^ DNA (20 ng) and ultra-pure water (QIAGEN, Brazil) were used. The conditions for amplification were: 1 cycle of 95°C for 5-min., followed by 40 cycles of 95°C for 10 sec., and annealing temperature of 60°C for 30 sec. After cycling, HRM was performed at temperatures from 75 to 95°C for 2 seconds at each temperature. As a negative control, a reaction mixture was used, replacing DNA with ultrapure water.

**Table 1:** Oligonucleotide sequences used to detect the polymorphisms evaluated.

**Polymorphisms (rs)**	**Oligonucleotides (5′‒3′)**	**HRM temperature (°C)**	**Products (pb)**	**Reference**
Progin +331 G >A (rs10895068)	F: CAGTGGAACTCAGCGAGGGAC	70 to 90	106	O'Mara et al.^18^
	R: TGCGACGGCAATTTAGTGACA			
V660L G >T (rs1042838)	F: TTCAATAAAGTCAGAGTTGTGAGAGCA	70 to 90	89	O'Mara et al.^18^ and Pooley et al.^19^
	R: TGGCTTAGGGCTTGGCTTT			

F, Forward; R, Reverse. A, Adenine, C, Cytosine, T, Thymine, G, Guanine.

Genotyping quality-control procedures included a no-template negative control in every HRM run, visual inspection of melting profiles in RotorGene *Q* Series software, and assignment of genotype clusters using expected wild-type, heterozygous, and homozygous-variant curve patterns. Exactly 309 samples were analyzed, all produced a final genotype for each variant, and no sample failed, corresponding to final analytic call rates of 100% for +331G/A and V660L. Systematic duplicate genotyping was not performed. Hardy-Weinberg equilibrium was assessed in controls as an additional quality-control check.

The HRM reactions were performed in a Rotor Gene 6000 thermocycler (Qiagen, Brazil) and analyzed in the RotorGene Q Series Software (Qiagen, Brazil). The HRM curves were analyzed according to the genotypes (wild-type, heterozygous and mutated) of each assay being the normalizers. As an endogenous control for the samples, the beta-globin gene was used. Detection assays were performed with the TaqMan system. Briefly, final volumes of 20 µL, GoTaq Probe qPCR MasterMix (Promega, Brazil), each pair of primers (10 µM), 10 µM TaqMan probe (Applied Biosystems, Foster City, CA) (Table 1), DNA (2 ng), and ultra-pure water (Promega, Brazil). The amplification conditions were: 1 cycle of 95°C for 2-min., followed by 40 cycles of 95°C for 5 sec., and 60°C for 30 sec. All amplifications were performed in a Rotor Gene 6000 thermocycler (Qiagen, Brazil).

### Sample size and statistical analysis

No formal a priori sample-size calculation was performed; the sample comprised the eligible participants included during the study period (236 EP+ cases and 73 EP-controls). Using the observed control carrier frequencies (9/73, 12.3%, for +331G/A; 20/73, 27.4%, for V660L), a two-sided exact test with alpha = 0.05 indicated 80% power to detect odds ratios of approximately 2.74 and 2.26, respectively (or inverse associations of approximately 0.21 and 0.38). These minimum detectable effect sizes show that small-to-moderate associations could not be excluded. The calculation was used to characterize precision rather than to reinterpret observed p-values.

Continuous variables were summarized as mean (SD) and range, and categorical variables as n (%). Group comparisons used Welch's *t*-test for continuous variables and Pearson's Chi-Square or Fisher's exact test for categorical variables, as appropriate. The two prespecified primary genetic comparisons were the carrier analyses for +331G/A and V660L. Because the study was exploratory, unadjusted two-sided p values and 95% Confidence Intervals are reported and secondary clinical comparisons are interpreted descriptively. As a multiplicity sensitivity analysis, a Bonferroni threshold of 0.025 was considered for the two primary genetic tests; this did not change the conclusions. Multivariable logistic regression used a binomial GLM with logit link and EP status as the dependent variable. Covariates were selected a priori based on clinical relevance and availability: age, BMI, operationally defined hypertension, diabetes mellitus, tamoxifen use, binary HT use, and carrier status for each PGR variant.

Serum E1 and E2 were measured in all 309 participants using blood collected from the arm on the day of hysteroscopy, before the procedure, according to the same protocol. Measurements were summarized descriptively but were not included in the primary multivariable models because they may lie on the causal pathway between hormone exposure and endometrial response rather than functioning solely as baseline confounders.

Adjusted Odds Ratios (aORs) with 95% Confidence Intervals are reported. Because homozygous variant genotypes were rare, genetic associations were evaluated using a prespecified carrier coding scheme (GA/AA vs. GG for +331G/A; GT/TT vs. GG for V660L). Hardy-Weinberg equilibrium in the EP-group was assessed using an exact test. Hypertension was defined as systolic blood pressure ≥ 140 mmHg and/or diastolic blood pressure ≥90 mmHg based on a single arm measurement obtained before the procedure, or current use of antihypertensive medication regardless of the measured blood pressure. Analyses were performed using *R* (R Foundation for Statistical Computing, Vienna, Austria).

### Reporting guideline

This study was reported in accordance with the Strengthening the Reporting of Observational Studies in Epidemiology (STROBE) statement.

## Results

All participants were postmenopausal. Baseline characteristics are shown in Table 2. E1 and E2 measurements were available for all 236 EP+ and 73 EP- participants. No statistically significant differences were observed for age, BMI, diabetes mellitus, dyslipidemia, smoking status, tamoxifen use, systolic or diastolic blood pressure, E1, or E2. Current HT use was more frequent in the EP- group than in the EP+ group.

**Table 2:** Baseline clinical characteristics of postmenopausal women with and without endometrial polyps.

**Variable**	**EP+ (n = 236)**	**EP- (n = 73)**	**p-value**
Ethnicity: White, n (%)	145 (61.45)	45 (61.65)	0.933
Ethnicity: Other, n (%)	91 (38.55)	28 (38.35)	0.933
Diabetes mellitus, n (%)	42 (17.80)	12 (16.44)	0.799
Dyslipidemia, n (%)	43 (18.20)	15 (20.55)	0.649
Current smoking, n (%)	13 (5.50)	6 (8.22)	0.441
Past smoking, n (%)	38 (16.10)	11 (15.07)	0.814
Tamoxifen use, n (%)	4 (1.70)	3 (4.10)	0.237
Hormone therapy use, n (%)	9 (3.81)	12 (16.44)	<0.001
Age, years; mean (SD) [range]	62.29 (7.34) [45‒83]	62.04 (7.77) [45‒80]	0.800
BMI, kg/m^2^; mean (SD) [range]	29.06 (5.08) [18.13‒48.28]	28.08 (4.97) [14.69‒41.09]	0.160
Pregnancies; mean (SD) [range]	3.29 (2.10) [0‒13]	3.24 (2.00) [0‒10]	0.866
SBP, mmHg; mean (SD) [range]	141.63 (18.63) [90‒222]	140.95 (16.24) [97‒179]	0.782
DBP, mmHg; mean (SD) [range]	80.97 (11.73) [46‒107]	82.80 (13.14) [42‒114]	0.263
E1, pg/mL; mean (SD) [range]	53.67 (35.95) [7.40‒195.10]; n = 236	53.47 (31.68) [6.40‒113.60]; n = 73	0.971
E2, pg/mL; mean (SD) [range]	11.30 (8.09) [5.00‒44.33]; n = 236	13.62 (16.39) [5.00‒93.70]; n = 73	0.182

Notes: Continuous variables are reported as mean (SD) [range]; categorical variables as n (%). Welch's *t*-test was used for continuous variables and Fisher's exact test for categorical variables. E1 and E2 were measured in all participants using a standardized blood collection performed before hysteroscopy. BMI, Body Mass Index; DBP, Diastolic Blood Pressure; E1, Estrone; E2, Estradiol; EP, Endometrial Polyp; HT, Hormone Therapy; SBP, Systolic Blood Pressure.

Genotype distributions for +331G/A and V660L did not differ significantly between groups (Table 3). For +331G/A, genotypes were GG 93.6%, GA 5.5%, and AA 0.8% in EP+ women and GG 87.7%, GA 11.0%, and AA 1.4% in EP- women (genotype comparison *p* = 0.245). The carrier comparison (GA/AA vs. GG) was not significant (OR = 0.48; *p* = 0.130). For V660L, genotypes were GG 79.7%, GT 19.1%, and TT 1.3% in EP+ women and GG 72.6%, GT 26.0%, and TT 1.4% in EP- women (genotype comparison *p* = 0.435). The carrier comparison (GT/TT vs. GG) was not significant (OR = 0.68; *p* = 0.201). Genotype counts totaled 309 at both loci, corresponding to final analytic call rates of 100%. Control distributions were consistent with Hardy-Weinberg equilibrium. Applying a Bonferroni threshold of 0.025 to the two primary carrier tests did not change either conclusion.

**Table 3:** PGR +331G/A and V660L genotype distributions and carrier analyses.

**Genotype**	**+331 EP+**	**+331 EP-**	**V660L EP+**	**V660L EP-**
GG	221 (93.6%)	64 (87.7%)	188 (79.7%)	53 (72.6%)
GA / GT	13 (5.5%)	8 (11.0%)	45 (19.1%)	19 (26.0%)
AA / TT	2 (0.8%)	1 (1.4%)	3 (1.3%)	1 (1.4%)

Notes: Genotypes are shown as n (%). Final genotype counts totaled 309 for each locus, corresponding to analytic call rates of 100%. For +331G/A, genotype comparison *p* = 0.245 and carrier comparison (GA/AA vs. GG) OR = 0.483, *p* = 0.130. For V660L, genotype comparison *p* = 0.435 and carrier comparison (GT/TT vs. GG) OR = 0.677, *p* = 0.201. Control distributions were consistent with Hardy-Weinberg equilibrium. A Bonferroni sensitivity threshold of 0.025 for the two primary carrier comparisons did not change the conclusions. V660L (rs1042838) proxies the PROGINS haplotype.

In multivariable logistic regression analysis adjusting for age, hypertension, DM, BMI, tamoxifen use, and HT use (Model 1, Table 4), neither +331G/A nor V660L carrier status was independently associated with EP (aOR = 0.43, 95% CI 0.17–1.09; *p* = 0.074 and aOR = 0.59, 95% CI 0.31–1.11; *p* = 0.100, respectively). Current HT use was less frequent in the EP+ group and remained inversely associated with EP in adjusted analysis (aOR = 0.15, 95% CI 0.06–0.40; p < 0.001); however, HT was recorded only as a binary exposure, without information on formulation, dose, route, duration, schedule, or indication.

**Table 4:** Multivariable logistic regression models for EP status (EP+ vs. EP-).

**Predictor**	**Model 1 aOR (95% CI)**	**p-value**	**Model 2 aOR (95% CI)**	**p-value**	**Model 3 aOR (95% CI)**	**p-value**
Age (per year)	1.029 (0.988‒1.071)	0.166	1.024 (0.984‒1.066)	0.241	1.028 (0.989‒1.068)	0.165
Hypertension	0.595 (0.324‒1.095)	0.095	0.610 (0.334‒1.113)	0.108	0.597 (0.327‒1.090)	0.093
Diabetes mellitus	1.18 (0.62‒2.25)	0.610	1.21 (0.64‒2.31)	0.566	1.17 (0.61‒2.23)	0.639
BMI	1.012 (0.965‒1.061)	0.627	1.009 (0.962‒1.058)	0.685	1.011 (0.964‒1.060)	0.648
HT use	0.154 (0.058‒0.405)	<0.001	‒	‒	0.159 (0.061‒0.415)	<0.001
Tamoxifen use	0.320 (0.068‒1.512)	0.150	0.362 (0.079‒1.655)	0.191	0.323 (0.069‒1.514)	0.151
+331G/A carrier	0.431 (0.171‒1.086)	0.074	0.427 (0.174‒1.049)	0.064	‒	‒
V660L carrier	0.587 (0.312‒1.106)	0.100	0.630 (0.341‒1.164)	0.140	‒	‒

Model 1: age, hypertension, diabetes mellitus, BMI, tamoxifen, HT, +331G/A carrier, and V660L carrier. Model 2: Model 1 excluding HT. Model 3: clinical covariates only (age, hypertension, diabetes mellitus, BMI, tamoxifen, and HT).

Notes: aOR, adjusted Odds Ratio; BMI, Body Mass Index; CI, Confidence Interval; EP, Endometrial Polyp; HT, Hormone Therapy. Secondary model p-values are interpreted descriptively. Hypertension was defined as systolic pressure ≥ 140 mmHg and/or diastolic pressure ≥ 90 mmHg based on a single arm measurement before the procedure, or current antihypertensive use regardless of measured pressure.

In Model 2, which excluded HT, both genetic associations remained non-significant. Model 3 was restricted to clinical covariates and reproduced the inverse association with the binary HT variable. All secondary model p-values are interpreted descriptively in view of the exploratory analyses and multiple comparisons.

Overall, neither evaluated PGR variant showed a statistically significant association with EP in unadjusted or adjusted analyses. Although some point estimates were below unity, confidence intervals crossed 1.0 and should not be interpreted as evidence of association.

## Discussion

In this exploratory case-control study of postmenopausal women undergoing hysteroscopy, we did not observe statistically significant associations between the PGR variants +331G/A or V660L and the presence of EP. This finding was consistent across genotype-based comparisons, carrier-based analyses used because of sparse homozygous-variant counts, and multivariable logistic regression models adjusting for available clinical covariates.

Although adjusted point estimates were below 1.0, their confidence intervals included 1.0. The results therefore do not establish either risk or protective genetic associations. The detectable-effect-size analysis further indicates that the study was unable to exclude small-to-moderate effects.

From a biological perspective, EP pathogenesis has been linked to dysregulated endometrial proliferation and tissue-level alterations in hormone receptor expression.^1^^,^^7^^,^^8^ In this context, PGR genetic variation has been considered a biologically plausible susceptibility factor for proliferative endometrial conditions. The PROGINS haplotype, commonly proxied by V660L (rs1042838), has been associated with diminished progesterone responsiveness in functional studies,^15^ but epidemiological associations across gynecologic phenotypes remain inconsistent.^13^^,^^14^ Similarly, the promoter variant +331G/A (rs10895068) has been described as functional and has been examined in hormone-related endometrial outcomes.^16^^,^^17^ Our findings do not support a statistically significant association between these variants and EP in this cohort, but the limited sample size prevents exclusion of small genetic effects.

The genetic analysis was constrained by the 236:73 case-control imbalance and low carrier frequency. With 80% power and two-sided alpha = 0.05, the minimum detectable ORs were approximately 2.74 for +331G/A and 2.26 for V660L. Associations smaller than these values could have remained undetected; the non-significant findings should therefore not be regarded as proof of absence.

Current HT use was less frequent among cases and inversely associated with EP after adjustment. This observation is hypothesis-generating, not evidence that HT prevents EP. HT was not suspended for hysteroscopy, but formulation, dose, route, duration, schedule, and indication were not collected. Selection into hysteroscopy, confounding by indication, prescribing patterns, and residual confounding could account for the association.

Study strengths include a single postmenopausal clinical setting, histopathological confirmation of cases, hysteroscopic confirmation of control status, complete final genotype calls in the analytic cohort, predefined carrier analyses, and explicit reporting of detectable effect sizes.

Limitations include the single-center observational case-control design, unequal group sizes, clinically selected rather than population-based controls, and the absence of individual matching. Although all eligible hysteroscopy-negative women during the study period were included and no participant declined, the distribution of referral indications and a numerical screening flow could not be reconstructed. HT and tamoxifen were recorded as current binary exposures without detailed dose, duration, schedule, or cumulative exposure information. Hypertension was based on a single preprocedural measurement or current antihypertensive use. Systematic duplicate genotyping was not performed, although all 309 samples yielded final calls and control genotypes were consistent with Hardy-Weinberg equilibrium. These factors limit causal interpretation and generalizability.

## Conclusions

In this exploratory case-control study, +331G/A and V660L were not statistically significantly associated with EP, but the sample had 80% power only for relatively large effects; smaller associations cannot be excluded. Current HT use was inversely associated with EP; this finding is hypothesis-generating and does not support causal inference because regimen-level exposure data were not collected, and selection or residual confounding may explain the association. Larger studies should use prospectively defined control sampling, detailed hormonal and tamoxifen exposure ascertainment, standardized hypertension assessment, and replicated genotyping quality control.

## Authors’ contributions

Conceptualization: MW, EO; Data curation: MW, MMS, MMACM, MFO; Investigation: MW, MT, MMS, MMACM, MFO, TM, RCMS; Project administration: CEF; Supervision: EO; Writing-original draft: MW; Writing-review & editing: EO.

## Funding

This study received in-kind laboratory support from Salomão & Zoppi Laboratory for the analysis of genetic variants. The supporter had no role in study design, data collection, statistical analysis, data interpretation, manuscript preparation, or the decision to submit the manuscript for publication.

## Data availability statement

De-identified participant-level data underlying the reported results and the corresponding data dictionary are available from the corresponding author upon reasonable request, subject to authorization by the participating hospital and applicable ethics, privacy, and consent requirements.

## Declaration of competing interest

The authors declare that they have no competing financial interests or personal relationships that could have appeared to influence the work reported in this manuscript.
